# Stem Cell-Derived Nanovesicles: A Novel Cell-Free Therapy for Wound Healing

**DOI:** 10.1155/2021/1285087

**Published:** 2021-09-14

**Authors:** Jianghong Huang, Jun Zhang, Jianyi Xiong, Shuqing Sun, Jiang Xia, Lei Yang, Yujie Liang

**Affiliations:** ^1^Department of Orthopedics, Shenzhen Second People's Hospital (First Affiliated Hospital of Shenzhen University, Health Science Center), Shenzhen 518035, China; ^2^Tsinghua University Shenzhen International Graduate School, Class 9 of 2020 Engineering Ph.D., Shenzhen 518055, China; ^3^Tsinghua University Shenzhen International Graduate School, Institute of Biomedicine and Health Engineering, Shenzhen 518055, China; ^4^Department of Chemistry, The Chinese University of Hong Kong, Sha Tin, Hong Kong SAR, China; ^5^Department of Child and Adolescent Psychiatry, Shenzhen Kangning Hospital, Shenzhen Mental Health Center, Shenzhen Key Laboratory for Psychological Healthcare & Shenzhen Institute of Mental Health, Shenzhen 518020, China

## Abstract

Wound healing and regeneration are a dynamic and complex process that requires a collaborative effort between growth factors, epidermal cells, dermal cells, extracellular matrix, and vessels local to the wound area. Mesenchymal stem cells participate in the recruitment site, mainly by releasing secretory factors and matrix proteins to promote wound healing. Stem cell-derived nanovesicles (CDNs), including microvesicles, exosomes, and exosome mimetics, contain most of the biologically active substances of their parent cells and have similar effects. CDNs can shuttle various proteins, messenger RNAs, and microRNAs to regulate the activity of receptor cells, and they play important roles in skin wound healing. This article reviews recent research progress on CDNs for wound repair. We summarize current knowledge on how CDNs regulate immunity, fibroblast activity, angiogenesis, and scar formation in the wound healing process. This review can help researchers explore new treatment strategies to enhance the therapeutic efficacy of CDNs, which have a promising future as naturally cell-free therapies.

## 1. Introduction

Approximately 100 million people suffer pain or discomfort from chronic wounds each year. Clinically, chronic wounds are common in large-area burns, late residual wounds, diabetic foot ulcers, venous leg ulcers, and pressure ulcers. The mildest wound is limited to the epidermis of the skin, while a more severe wound breaks the skin and subcutaneous tissue, and severe trauma can fracture the muscle, muscle bonds, and nerves. Wound healing refers to the healing process after the skin tissue is damaged by external forces and includes the complex, synergistic combination of tissue regeneration, granulation tissue hyperplasia, and scar tissue formation.

**Figure 1:**
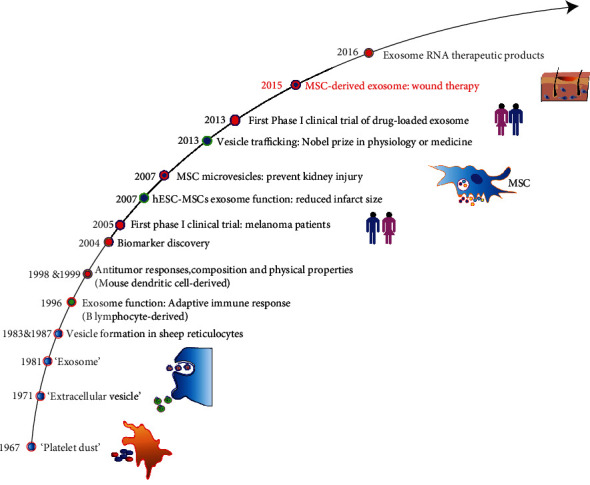
Timeline of the discovery and application of CDNs.

The development and application of tissue-engineered skin for wound healing has made significant progress. Some skin grafts and skin substitutes have been used clinically; however, they cannot meet the needs of patients with severe skin defects. In recent years, many researchers have applied mesenchymal stem cells (MSCs) as a type of pluripotent stem cell with self-renewal and multidirectional differentiation capabilities. Extracellular microvesicles (EVs) and exosomes (Exo) derived from MSCs are highly enriched in secreted bioactive factors. MSC-derived nanovesicles have been proposed as a new “cell-free” treatment for skin wounds [1]. Compared with MSCs, cell-derived nanovesicles (CDNs) have shown not only higher therapeutic efficiency but also more convenient preparation, storage, transport, and administration. Moreover, they avoid the risk of immune rejection and tumorigenesis that come with stem cell transplantation. Therefore, MSC-derived nanovesicle-mediated therapy may be safer and more efficient than MSC-based therapy. Since 2015, many studies have explored CDNs for skin wound healing [2, 3] (Table 1 and Figure 1).

In this review, the roles of CDNs in skin wound healing are overviewed. In particular, the important roles of CDNs in each phase of the wound healing process are described. The mechanisms of action of CDNs are further discussed. The use of CDNs in combination with hydrogels for the treatment of skin wounds is also described.

## 2. Biological Characteristics of CDNs

**Figure 2:**
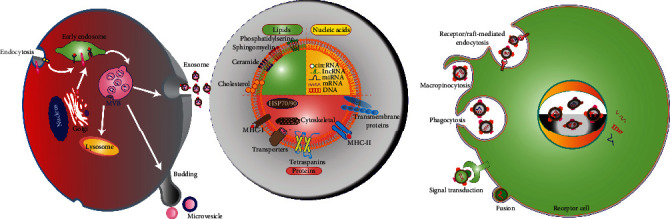
Schematic diagram of CDN biogenesis, structural composition, and cellular internalization. Exos are formed following plasma membrane invagination to form MVBs that are then exocytosed. The main components of CDNs are lipids, proteins, and nucleic acids. CDN lipids include cholesterol, ceramides, and phosphatidylserines, while CDN proteins include ubiquitous tetraspanins (CD9, CD63, and CD81) and HSPs. CDNs can (i) trigger cell signal transduction through intracellular signaling pathways and release of secretory contents; (ii) fuse with cell membranes, transfer proteins and genetic content, and enter the cytoplasm of recipient cells; and (iii) be endocytosed through phagocytosis, macropinocytosis, or receptor-mediated endocytosis.

Exos, EVs, and Exo mimetics are three types of nanoscale membrane vesicles that originate from native cells (Figure 2). Exos are secreted into the extracellular environment by a variety of cell types and tissues. The membrane surface markers of Exos mainly include CD9, CD63, CD81, and heat shock protein 70 (HSP70). Negative staining electron microscopy has shown that Exos isolated from different body fluids have a high degree of morphological diversity. Exos are cup-shaped under transmission electron microscopes, while they have a round morphology under cryoelectron microscopes. Immunogold labelling of Exos has been used to show the location of positive proteins (e.g., CD63) on Exos.

The biogenesis of Exos is a strict and refined regulation process that is divided into 3 stages: (1) deformation and invagination of the plasma membrane, (2) encapsulation of intracellular material to form vesicles and gradual fusion of the plasma membrane to support the formation of multivesicular bodies (MVBs), and (3) exocytosis of MVBs to form Exos. EVs (100–1000 nm in diameter) are secreted by shedding of the plasma membrane or outward budding. Exo mimetics are a type of nanovesicle with a similar size, morphology, and membrane protein labeling to native Exos. High-activity Exo analogs can be produced in large quantities by continuous extrusion.

Most cells and tissues can release CDNs, including MSCs, oligodendrocytes, B cells, T cells, endothelial cells, epithelial cells, tumor cells, adipose tissue, and cartilage tissue. CDNs carry DNA, microRNAs (miRNAs), proteins, cytokines, and membrane lipids from their parent cells, and they can release, transport, and transfer these molecules to recipient cells (such as endothelial cells, keratinocytes, and fibroblasts) to regulate a variety of biological processes, including inflammation, cellular immunity, cell proliferation, cell apoptosis, cell migration, and vascularization [4]. The surface of CDNs contains various lipids, including diglycerides, cholesterol, sphingolipids, and phospholipids, that maintain their nanostructure. The lipid bilayer membrane structure facilitates delivery of the vesicles to target cells and protects their protein, cytokine, and RNA cargo from degradation. In addition, the lipid component includes signaling molecules that can participate in various cellular responses. CDNs contain a variety of proteins and genetic material that play important roles in their therapeutic effects. In terms of their protein component, CDNs contain proteins that are involved in angiogenesis and coagulation processes [5]. Exosomal matrix metalloproteinase (MMP) has been identified as an important mediator of angiogenesis [6, 7]. Fibroblast growth factor 2 (FGF2), vascular endothelial growth factor (VEGF), hepatocyte growth factor (HGF), and platelet-derived growth factor BB (PDGF-BB) have been identified in Exos and can stimulate angiogenesis [7]. In addition, tissue factor (TF) from platelets and monocytes has been detected in EVs, and TF is an important substance in the exogenous coagulation cascade [8]. Exos derived from dendritic cells contain major histocompatibility complex (MHC) class I molecules that can activate the response of CD8+ T cells, suggesting that EVs may serve as a source of extracellular antigens to activate immune intervention, enhance the antibacterial ability of wounds, and ultimately promote wound healing [9]. In terms of genetic biomolecules, a large number of messenger RNAs (mRNAs) and miRNAs carrying information from the parent cells have been found in EVs released in cell culture and body fluids. These genetic molecules are sorted and secreted by CDNs and then act on target cells to promote a variety of biological functions, such as angiogenesis and tissue repair. It has recently been discovered that MSC-derived EVs can mediate mRNA delivery to regulate the transcription of tissue-specific mRNAs, indicating that the mRNA present in stem cell-derived nanoparticles has a regulatory function [10]. Deregibus et al. found that EVs derived from endothelial progenitor cells activate endothelial cell angiogenesis through horizontal mRNA transfer [11]. In addition, there is evidence that EV mRNA can be translated into protein after being transferred to recipient endothelial cells, which was shown to activate the phosphoinositide 3 kinase (PI3K)/protein kinase B (AKT) signaling pathway and accelerate angiogenesis [11, 12]. The above studies provide evidence for the roles of EV mRNA and miRNA in the regulation of biological processes, but further research is still needed to fully understand the roles of EVs as gene carriers.

CDNs can act on recipient cells in a variety of ways. Through receptor-ligand interactions, binding of CDNs to cell surface receptors activates signaling pathways that mediate internalization by endocytosis [13]. CDNs can also be internalized through phagocytosis, pinocytosis, and micropinocytosis [14]. Their information-rich cargoes are then transmitted to the target cells to regulate the microenvironment and related protein functions [15].

## 3. MSC-Derived Nanovesicles in Wound Healing

MSCs have the potential for multidirectional differentiation, self-replication, and renewal, and they are often used as seed cells in the field of tissue regeneration [16]. MSCs can be obtained from many adult tissues. The most used sources of MSCs are bone marrow and adipose tissue [17, 18]. They can also be separated from a range of other tissues, including Wharton's glue, cord blood [19], placenta [20], brain [21], and synovial fluid [22, 23]. Nearly a dozen studies have confirmed that MSCs play a positive role in wound repair. They participate in the entire process of wound repair and exert a variety of biological effects. Intradermal injection of MSCs is a common method that can promote skin damage repair, but the area and depth of the injection cannot be controlled and it may also cause additional tissue damage. As injected MSCs lack adhesion to the extracellular matrix (ECM), they suffer from poor cell persistence and survival upon engraftment [24]. These problems limit the ultimate therapeutic potential of MSCs for wound repair.

**Figure 3:**
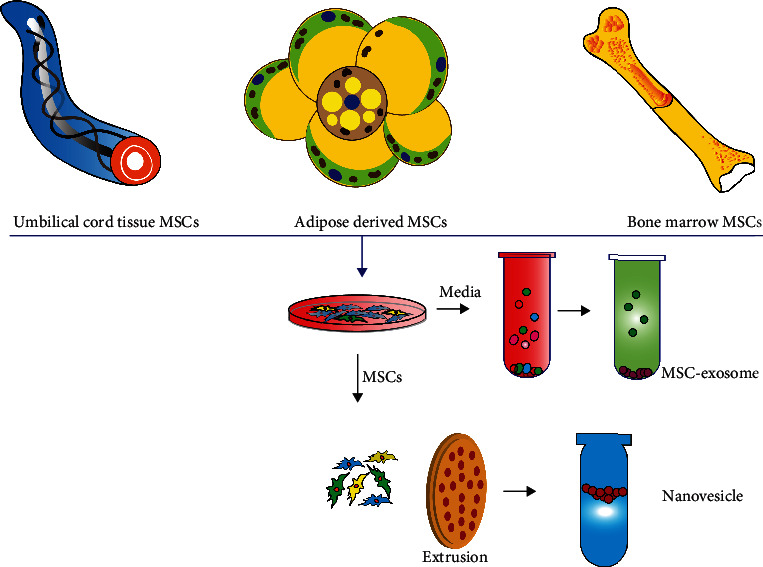
MSCs derived nanovesicles as a cell-free therapy for wound repair.

**Figure 4:**
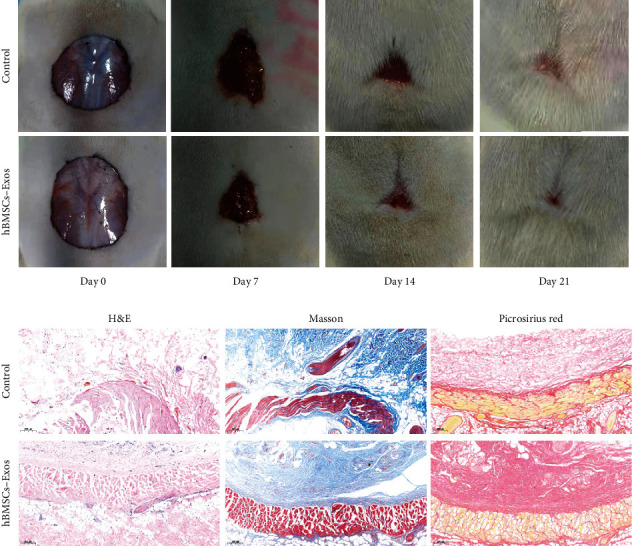
The representative photos shown of full-thickness excision wound area of the rat treated with PBS (control) or hBMSCs-Exos. Reproduced with from Ref. [69]. Copyright 2021 BioMed Central Ltd.

Compared with MSCs, MSC-derived nanovesicles have several obvious advantages. First, CDNs can directly fuse with target cells and exert a strong biological effect. Second, their cargo is protected from degradation by the lipid bilayer. Third, the concentration, dosage, and route of administration of CDNs is more controllable. Fourth, CDN therapy can avoid the risks of immune rejection and tumor occurrence that may be caused by the cell transplant. Therefore, MSC-derived nanovesicles can be used as an alternative to MSCs in the future. Researchers have reported applications of MSC-derived nanovesicles for wound repair in rat and mouse models since 2015 (Figure 3). In Table 1, we have summarized the research on the use of MSC-derived nanovesicles for wound healing. The supernatant of cell culture was used to collect CDNs. Usually, ultracentrifugation was used for purification of CDNs, but the yield is low. Other additional purification steps, including size exclusion chromatography and sucrose or OptiPrep density gradients showed better yield and purity. Standardization is essential for the application of CDNs. Characterization of CDNs includes western blotting, liquid chromatography, and mass spectrometry for proteomic analysis. The size and concentration of exosomes were assessed by nanoparticle tracking analysis, the morphology was characterized by transmission electron microscopy, and CDN surface analysis can use the flow cytometry technique. Standardization of the storage method of CDN components is also a key step. Storage at 4°C or -20°C will affect the biological activity and protein content of CDNs. It is generally recommended to store at -80°C which is the optimal temperature that has the least effect on the morphology and content of exosomes. We also used a model with a full-thickness incision wound to prove that exosomes derived from hBM-MSC can accelerate the skin wound healing process *in vivo* (Figure 4).

## 4. Mechanisms of MSC-Derived Nanovesicles in Wound Healing

**Figure 5:**
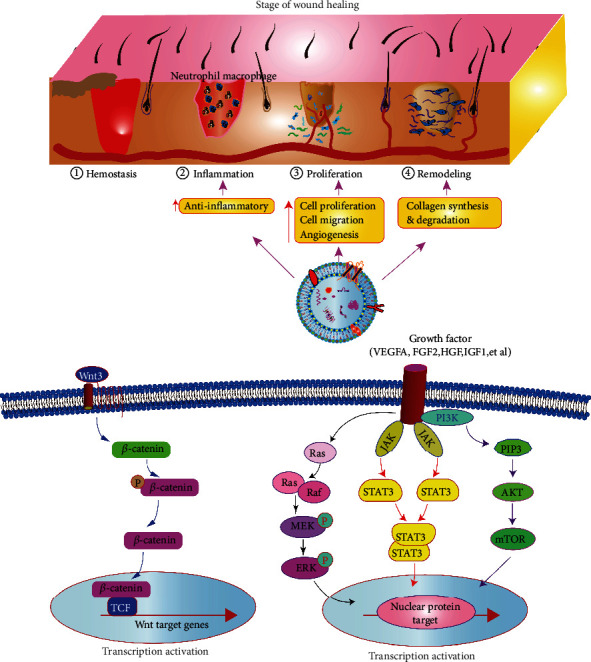
CDNs promote wound healing by enhancing angiogenesis, reducing inflammation, increasing cell proliferation and migration, and restoring tissue integrity. The contribution of signaling pathways in which the exosome can activate the Wnt3/catenin, PI3K/Akt, JAK/STAT3, Ras/ERK signaling pathways to further accelerate wound healing.

Wound healing is a highly organized multistep process that restores tissue integrity after injury. It involves interactions between various cell populations and is usually divided into four overlapping stages: hemostasis, inflammation, proliferation, and remodeling [25]. MSC-derived nanovesicles can regulate wound repair during inflammation, cell migration, cell proliferation, angiogenesis, collagen production, and ECM remodeling. At present, CDNs are known to activate a variety of important signaling cascade pathways related to the final three stages of wound repair (Figure 5), including AKT, extracellular signal-regulated kinase (ERK), signal transducer and activator of transcription 3 (STAT3), and Wnt/*β*-catenin [2, 26, 27]. CDNs can also stimulate several signaling pathways to induce the expression of several important growth factors related to wound repair, such as insulin-like growth factor 1 (IGF1), HGF, and stromal cell-derived factor 1 (SDF1) [27]. These growth factors can promote angiogenesis, cell migration, cell proliferation, and reepithelialization.

### 4.1. CDN Function in Hemostasis

Studies have revealed that the main therapeutic effect of platelets during bleeding is dependent on the effect of platelet-derived extracellular vesicles [28]. Neonatal plasma exosomes can be enriched in a large number of proteins involved in platelet function and primary hemostasis, such as platelet activation and signaling proteins, integrins *α*IIb and *β*3, platelet chemotactic protein (PF4), filaggrin-A, and ligand receptor (CD36) [29]. The use of platelet exosomes shows the advantage of the hemostatic effect over fresh platelets [30]. Platelet exosomes can improve the outcome of severe trauma by maintaining hemodynamic stability and reducing the development of ischemia and metabolic acidosis, which provide a prohemostatic support for the early wound stage.

### 4.2. CDN Function in Inflammation

Inflammation is a self-defense mechanism against wound injury that occurs within 24–48 h of ischemia onset. Acute and regulated inflammation can promote wound healing and epithelial regeneration [31]. MSC-derived nanovesicles can downregulate proinflammatory enzymes, such as inducible nitric oxide synthase (iNOS) and cyclooxygenase (COX2), as well as secreted cytokines and chemokines to reduce inflammation during wound repair [32]. In addition, MSC-derived nanovesicles can upregulate the anti-inflammatory cytokine interleukin 10 (IL10), which has been reported to play a vital role in the control of skin wound inflammation and scar formation [33]. Macrophages are prominent inflammatory cells that play an important role in the process of skin regeneration. MSC-derived nanovesicles can promote the significant switch of macrophages to the anti-inflammatory M2 phenotype [34]. Additionally, MSC-derived Exos rely on the Janus kinase 2 (JAK2)/STAT6 pathway to mediate macrophage activation, which can significantly reduce the number of proinflammatory macrophages [35]. Lipopolysaccharide- (LPS-) pretreated MSC-derived Exos have been shown to regulate macrophage polarization and chronic inflammation. Exo-specific let-7b released by LPS-pretreated MSCs can stimulate the toll-like receptor 4 (TLR4) pathway, inhibit inflammation, and promote normal wound healing [36]. In addition, the immunomodulatory effect of human umbilical cord-derived MSCs (hUC-MSCs) preactivated with IL1*β* is partly caused by Exo-mediated miR-146a transfer [37]. Melatonin-pretreated MSC-derived Exos can increase M2 polarization relative to M1 polarization by upregulating the expression of phosphatase and tensin homolog (PTEN) and inhibiting phosphorylation of AKT, which can significantly inhibit the proinflammatory factors IL1*β* and tumor necrosis factor alpha (TNF*α*) and increase the expression of the anti-inflammatory factor IL10. Thus melatonin-pretreated MSC-derived Exos can significantly facilitate diabetic wound healing [38]. These findings provide research directions for enhancing the anti-inflammatory functions of Exos. MSC-derived Exos can regulate the activation, differentiation, and proliferation of B lymphocytes and T lymphocytes. Moreover, MSC-derived Exos can transform activated T lymphocytes into a T regulatory phenotype, thereby exerting an immunosuppressive effect [39]. Studies have also found that MSC-derived Exos exert immunomodulatory effects through specific miRNAs. Three miRNAs (miRNA-21, miRNA-146a, and miRNA-181c) enriched in hUC-MSC-derived Exos were shown to be related to specific immune responses and inflammation regulation [40]. hUC-MSC-derived Exos overloaded with miR-181c were found to reduce burn-induced inflammation in rats by downregulating the TLR4 signaling pathway [41]. In general, further research is needed to clarify the specific molecular mechanisms underlying inflammation inhibition by MSC-derived nanovesicles during wound healing and skin regeneration.

### 4.3. CDN Function in Angiogenesis

Wound angiogenesis is one of the main mechanisms by which CDNs promote skin damage repair. Adequate wound blood provides nutrition, oxygen, and cell migration pathways for the regeneration of damaged tissues. MSC-derived Exos are rich in various proteins related to angiogenesis and miRNAs that activate multiple signaling pathways in endothelial cells. Previous research showed that MSC-derived nanovesicles contain a large number of angiogenic factors, such as VEGFA, FGF2, MMP, lactadherin (MFG-E8), angiopoietin-related protein 1 (ANGPTL1), and thrombopoietin. Recently, Zhang et al. reported that Exos derived from hUC-MSCs are rich in WNT4 protein, which can activate the Wnt/*β*-catenin signaling pathway to promote the reconstruction and regeneration of blood vessels after scalding wounds [42]. Exos derived from human umbilical cord blood-derived endothelial progenitor cells (EPCs) can activate the ERK1/2 signaling pathway to upregulate the expression of vascular genes, such as VEGFA, COX2, and FGF2, to promote cutaneous wound healing and regeneration [43]. Exos from human urine-derived stem cells are rich in deleted malignant brain tumor 1 (DMBT1), which has been shown to induce angiogenesis in cultured endothelial cells and promote angiogenesis and wound healing in diabetic mice [44]. In addition, the proangiogenic effect of EPC-derived Exos in endothelial cells may be partly attributed to inhibition of the MMP9 expression [45].

Previous studies have found that EVs are also enriched with a large number of miRNAs that promote angiogenesis. For example, fibrocyte-derived nanovesicles were found to be enriched with miRNA-126, miRNA-130a, and miRNA-132 during wound closure [46]. miR-125a is also enriched in Exos from adipose-derived MSCs (ADSCs), which were found to transfer miR-125a to endothelial cells for targeted knockdown of the angiogenesis inhibitor delta-like canonical Notch ligand 4 (DLL4) [47]. Additionally, exosomally transferred miR-21 can act on the target gene PTEN to activate the downstream mitogen-activated protein kinase (MAPK)/ERK and PI3K/AKT signaling pathways to promote vascularization [48].

Studies on the mechanisms of Exos in angiogenesis will help us better understand various physiological and pathological processes in wound repair. Although some studies have demonstrated the potential roles of exosomal miRNAs and protein factors, further research is needed to determine their overall importance compared to the broader secretory group and the mechanisms that underlie exosomal transport of specific cargo. Further research on miRNAs and proteins transferred from Exos may provide new directions for enhancing the therapeutic regulation of angiogenesis during wound healing.

### 4.4. CDN Function in Cell Proliferation and Migration

Cell proliferation and skin reepithelialization are essential for wound healing. Skin fibroblasts play an important role in the repair and regeneration of damaged tissue. The basic characteristics of wound proliferation include cell proliferation, migration of various cell types, and matrix protein synthesis. This stage is also described by the formation of a new tissue organization. Epithelial cells begin to migrate to the edge of the injured area, closing the edge of the wound, while matrix proteins provide an external environment to promote cell attachment to the scaffold. The synthesis and deposition of ECM play an important role in this stage. Under certain cell stimulation conditions, fibroblasts can differentiate into myofibroblasts, which play an important role in wound contraction.

MSC-derived Exos can be internalized *in vivo* and their contents (such as proteins and RNAs) transferred to receiving cells to regulate their proliferation and migration. It has been demonstrated that EVs from a variety of cell sources can accelerate the proliferation and migration of fibroblasts and keratinocytes [49]. For example, EVs from ADSCs and bone marrow-derived MSCs (BMSCs) have been shown to promote the growth and migration of fibroblasts from chronic diabetic ulcer wounds in a dose-dependent manner *in vitro* [46]. Exos from human-induced pluripotent stem cell- (hiPSC-) derived MSCs were also shown to promote the proliferation and migration of human dermal fibroblasts and epidermal cells in a dose-dependent manner *in vitro* [50]. Exos also promote the proliferation of skin cells and are associated with increased levels of cytokeratin 19 (CK19) and proliferating cell nuclear antigen (PCNA). For example, Exos derived from hUC-MSCs were shown to increase the expressions of CK19, PCNA, and collagen I, which accelerate wound healing and promote epithelial regeneration [2]. In addition, hUC-MSC-derived EVs were shown to inhibit the proliferation and apoptosis of keratinocytes and dermal fibroblasts by inhibiting the proapoptotic protein B cell lymphoma 2 (BCL2) and BCL2-like protein 4 (BAX). In a rat model study, it was found that ADSC-derived Exos can be internalized by fibroblasts, inducing skin wound repair and fibroblast activity. Results from this study showed that the gene expressions of N-cadherin, cyclin 1, PCNA, type I and type III collagen, and elastin were significantly increased in a dose-dependent manner under stimulation by ADSC-derived Exos [51]. It has been reported that the MAPK, Wnt, and mechanistic target of rapamycin (mTOR) pathways all play important roles in the process of wound healing. As a special intercellular signaling pathway, Exos have a common molecular mechanism that regulates the proliferation and migration behavior of skin cells, rather than skin cell specificity. Exos can induce important intracellular signaling pathways, including AKT, STAT3, Wnt, and ERK. These signaling pathways can upregulate various growth factors in target cells (such as HGF, IL6, IGF1, and nerve growth factor). For example, EVs derived from keratinocytes can upregulate the MAPK/ERK pathway. hUC-MSC-derived EVs were shown to activate Wnt/*β*-catenin in skin cells by transferring WNT4 to promote wound healing in a rat burn model [2]. BMSC-derived Exos also transport WNT3A to enhance fibroblast proliferation and migration [52]. Synovial MSC-derived EVs are enriched in miRNA-126-3p, which regulates the PI3K/AKT and MAPK/ERK pathways to induce the proliferation and migration of dermal fibroblasts [53].

These common signaling pathways provide the potential for applications of Exos in other fields of biology and the treatment of other diseases. Nevertheless, the components of Exos that play functional roles and the upstream and downstream phosphorylation regulation mechanisms in related molecular pathways still need to be investigated.

### 4.5. CDN Function in Wound Remodeling

ECM remodeling usually lasts from 2 weeks to one year or more after injury. The remodeling stage of wound healing is closely related to the production and reorganization of ECM, which is critical to scar formation. In the later stage of wound repair, related effector cells undergo apoptosis, type I collagen is replaced with type III collagen, MMPs degrade ECM, and other ECM proteins are synthesized. The key to ECM reconstruction is the synthesis and degradation of collagen. EVs regulate the final stage of wound healing by stimulating the secretion of ECM. In the early stage of wound healing, Exos derived from MSCs can increase the amount of collagens I and III in the wound bed. However, in the late stages of chronic wound healing, the effects of EVs shift to inhibition of collagen expression and fibroblast differentiation into myofibroblasts, which can inhibit scar hyperplasia [54]. EVs from fibroblasts can stimulate the synthesis of collagen in the matrix and enhance the deposition of mature collagen fibers in the wound area [46]. Exos derived from hiPSCs have been observed to enhance the synthesis of type I and III collagen in wound sites [50]. Another study showed that Exos from human amniotic epithelial cells regulate the ratio of collagens I and III by stimulating the expression of MMP1, which promotes wound healing and inhibits scar formation [55]. Intravenous injection of ADSC-derived Exos in full-thickness dorsal wound model mice was shown to inhibit the expressions of collagens III and I in fibroblasts by increasing the transforming growth factor beta 1 (TGF*β*1)/TGF*β*3 ratio, thereby preventing fibroblast differentiation into myofibroblasts and inhibiting the formation of granulation tissue, which reduced scar formation. In addition, ADSC-derived Exos were shown to activate the MAPK/ERK pathway in skin dermal fibroblasts *in vitro*, increase the ratio of MMP3 to TIMP1, and promote scarless skin repair by regulating ECM remodeling [51]. In addition, during skin regeneration, hUC-MSC-derived Exos effectively promoted the phosphorylation of yes-associated protein (YAP) by transporting the 14-3-3*ζ* protein, thereby mediating the binding of YAP and the phosphorylated large tumor suppressor (p-LATS). Phosphorylation of YAP also inhibited Wnt/*β*-catenin signal transduction, restricting excessive dermal fibroblast expansion and collagen deposition in burn wounds, which played a key role in improving tissue remodeling and reducing scar formation [49].

These studies provide strong *in vitro* and *in vivo* evidence that MSC-derived Exos play important roles in ECM remodeling during wound repair, indicating that Exos have clinical application prospects in tissue wound healing. It is important to note that the roles of Exos in all stages of wound healing are complementary. In addition, a larger range of prospective, blinded, randomized, and placebo-controlled clinical trials is needed to further verify the safety, effectiveness, and durability of Exos.

## 5. Discussion and Conclusion

In the past few decades, numerous studies have used stem cells and CDNs for wound repair and skin regeneration. Exo-based therapies are becoming a promising technology that can promote wound healing and minimize scar formation. As cell-free therapies, Exos have many advantages, such as ease of preparation, storage, transport, and administration. They also have demonstrated high therapeutic efficiency without risk of immune rejection and tumor development. MSC-derived Exos have significant wound regeneration potential and can effectively replace MSC-based therapies. The processes in each stage of wound repair are usually carried out in a precise and programmed manner involving a variety of intracellular and extracellular pathways, inflammatory pathways, the immune system, the coagulation cascade, and other complex processes. Thus, the participation of Exos in wound healing is very complicated. Further studies on the content and function of Exos can reveal the molecular mechanisms involved in wound repair and provide sufficient knowledge for the application of cell-free therapies for regenerative medicine. Currently, most of the mechanisms discovered have been studied in rodent models. However, animal physiology cannot always be translated to humans. Therefore, further clinical trials using human cell-derived Exos are needed to clearly demonstrate the therapeutic potential of Exos for wound healing.

Therapeutic applications of Exos are limited by their rapid clearance, short half-lives, and difficult large-scale preparation. Loading Exos into a supporting scaffold can prevent them from being quickly removed from the wound area. Additionally, synergies between Exos and scaffolds have been found that effectively promote wound repair [56, 57]. We have highlighted those studies that utilize Exos loaded in bioactive scaffolds to promote wound healing in Table 2. The application of CDNs in scaffold materials is still at an early stage. Studying the immune response of CDN-laden scaffolds in wound repair is a long-standing topic for the development of biocompatible biomaterial. The development of the active composition of CDN-laden scaffolds that appropriately interact with the immune system is facing a huge challenge. Studies have found that immune cells can absorb exosomes from the scaffold with more priority, which means that the body's immune response is important in tissue response [58].

Although CDNs have great advantages, they do not have the ability to target diseased organs after systemic injection, so that CDNs need multiple injections to achieve a certain therapeutic effect. In order to improve the targeting efficiency of CDNs to diseased organs, we have genetically engineered exosomes to improve the efficiency of targeted delivery for cartilage tissue repair [59–61]. These strategies can also be used to modify stem cells to obtain targeted stem cell-derived nanovesicles.

Although nanovesicles derived from different cell sources have different potentials, their physical and biological functions show high consistency. However, generation of CDNs is closely related to the microenvironment. Recent studies have also shown that the inflammatory microenvironment can significantly increase Exo release from MSCs. Interferon gamma (IFN*γ*) can enhance the immunomodulatory activity of hUC-MSC-derived Exos [62]. LPS pretreatment of hUC-MSCs was found to significantly alter Exo secretion, and Exos from pretreated cells were rich in the miRNA let-7b, which alleviated inflammation and enhanced wound healing [63]. Furthermore, other studies have shown that under a 1–2% hypoxic environment, TNF*α*-pretreated 3D culture systems have significantly altered MSC paracrine factor compositions. Therefore, the release and composition of CDNs can be adjusted by changing the cell culture microenvironment to enhance their wound repair functions. In addition, Exos can be loaded with therapeutic small molecule drugs or gene drugs to enhance their effects [64–66].

For extensive clinical application of Exos, there are still some urgent problems that need to be solved. At present, the extraction and purification of Exos is relatively cumbersome. It is not possible to quickly isolate the number of Exos required to meet clinical needs. There also is a lack of standardized methods for identifying Exos from specific cell sources, and proteomic detection and analysis of Exos are currently not available. Most importantly, the clinical implementation of any therapy must be based on safety. Further research is needed to determine the effective Exo dose for specific applications. Since research in the field of Exos is still in its infancy, the biological safety, effectiveness, reproducibility, production potential, formation mechanism, and biological functions of Exos that contribute to wound healing are still unclear. Despite the current challenges, the use of Exos for wound treatment is promising and inspiring. At present, more and more clinical treatment trials for CDNs are also underway, including for cancer, neurodegeneration, and infectious diseases. Stem cells are the main source for therapeutic CDNs, especially for regenerative medicine and immunomodulation. The phase I trial (NCT02138331), led by Nassar et al., uses CDNs derived from hUC-MSCs to ameliorate inflammatory immune reactions in chronic kidney diseases [67]. Previous clinical trials (NCT04276987, NCT04313647) used the immunomodulatory effects of CDNs from ADSCs for the treatment of lung injury [68]. Ongoing phase I clinical trials for the treatment of cutaneous wound healing use autologous exosomes from the participants' own plasma (NCT02565264). We highly expect that more exciting applications of Exos in clinical practice will emerge in the near future.

## Figures and Tables

**Table 1:** Overview of research on the use of CDNs for wound healing.

Cell source	Wound type	Function	Reference
LPS-pretreated hUC-MSCs	Diabetic cutaneous wound	Suppress inflammation and promote healing of diabetic wounds	[63]
H_2_O_2_-pretreated ADSCs	Skin flap model: ligation of blood vessels and blood perfusion	Improve the survival rate of animals with skin flaps; promote neovascularization and inhibit inflammation and cell apoptosis	[70]
Salidroside-pretreated mouse MSCs	Diabetic wound	Accelerate reepithelialization and wound closure	[71]
Pioglitazone-pretreated BMSCs	Diabetic wound	Promote angiogenesis and enhance wound repair	[72]
Neonatal serum-pretreated BMSCs	Skin wound healing model	Promote angiogenesis	[73]
Melatonin-pretreated hBMSCs	Rat diabetic wound healing model	Immunosuppressive effects	[38]
hUC-MSCs	Scratch assay and human skin permeation	Accelerate collagen synthesis and cell migration	[74]
hUC-MSCs	Rat burn injury model	Enhance wound closure and inhibit skin cell apoptosis	[2]
hUC-MSCs	Severe burn	Suppress inflammation and promote neovascularization	[75]
hUC-MSCs	Rat model of deep second-degree burn injury	Accelerate angiogenesis	[76]
hiPSCs	UV irradiation damage of human dermal fibroblasts	Ameliorate aging of skin fibroblasts	[77]
hiPSC-MSCs	Rat skin wound model	Promote collagen synthesis and enhance angiogenesis	[50]
Menstrual blood-derived MSCs	Diabetic foot ulcer	Suppress inflammation and accelerate wound healing	[78]
Human fibrocytes	Genetically diabetic ulcers	Enhance angiogenesis and augment keratinocyte proliferation and migration	[46]
Platelet-rich plasma	Rat diabetic wound model	Promote reepithelialization	[79]
Platelets	Ischemic wound healing	Regulate collagen synthesis and restore dermal architecture	[80]
Human dermal fibroblasts	UVB-induced skin photoaging	Reduce skin photoaging and inflammation	[81]
Human endothelial progenitor cells	Rat diabetic wound model	Regulate endothelial function	[82]
Human urine-derived stem cells	Mouse diabetic wound model	Promote the angiogenic activities of endothelial cells	[44]
Macrophage-derived Exos	Diabetic wound model	Promote endothelial cell proliferation, angiogenesis, and reepithelialization	[83]
Human amniotic epithelial cells	Rat full-thickness skin wound model	Stimulate fibroblast proliferation and migration; decrease scar formation	[55]
Oral mucosal epithelial cells	Rat full-thickness skin wound model	Proregenerative effects	[84]

Abbreviations: ADSC: adipose-derived MSC; BMSC: bone marrow-derived MSC; hiPSC: human-induced pluripotent stem cell; hUC-MSC: human umbilical cord-derived MSC; LPS: lipopolysaccharide.

**Table 2:** Summary of research on immobilized Exos in wound repair.

Exosome	Scaffold	Model	Function	Reference
hUC-MSC-derived Exos	Pluronic F127 hydrogel	Diabetic full-thickness skin wound	Accelerate wound closure and promote tissue regeneration	[85]
pH-responsive Exos	Polysaccharide-based dressing	Chronic diabetic wound	Enhance the angiogenic ability and sustained release of Exos	[86]
Mouse ADSC-derived Exos	Pluronic F127 hydrogel, oxidized hyaluronic acid, and *ε*-polylysine	Rat diabetic cutaneous injury	Sustained release of Exos; promote angiogenesis, reepithelialization, neovascularization, and cell proliferation	[87]
ADSC-derived Exos	Alginate hydrogel	Full-thickness excisional wound	Enhance wound closure, reepithelization, collagen deposition, and angiogenesis	[88]
ADSC-derived Exos	Porous cryogels	Diabetic and infectious wound	Accelerate wound closure, promote collagen deposition, increase reepithelialization and neovascularization, and decrease oxidative stress	[79]
hUC-MSC-derived Exos	Hydromatrix	Skin wound healing	Inhibit myofibroblast formation and reduce scar formation	[89]
hUC-MSC-derived Exos	Genipin-crosslinked hydrogel	Rat cutaneous wound	Reduce inflammation, promote wound closure, accelerate epithelial regeneration, and increase collagen deposition	[90]
Gingival MSC-derived Exos	Chitosan/silk-based hydrogel	Rat diabetic wound	Promote reepithelialization, deposition and remodeling of collagen, angiogenesis, and skin wound healing	[89]
BMSC-derived Exos	Electrospun fibrous scaffolds	Full-thickness skin wound	Synergistic immunomodulatory functions toward skin wound repair	[58]
ADSC-derived Exos	Pluronic F127 hydrogel, oxidized hyaluronic acid, and *ε*-polylysine	Diabetic full-thickness cutaneous wound	Sustained release of Exos; promote neovascularization, accelerate the formation of granulation tissue, and enhance reepithelialization and collagen remodeling	[91]
Placental MSC-derived Exos	Aldehyde methylcellulose–chitosan-*g*-PEG hydrogel	Full-thickness skin defect	Synergistically promote angiogenesis and inhibit cell apoptosis; promote the function of hair follicles and glands	[92]

Abbreviations: ADSC: adipose-derived MSC; BMSC: bone marrow-derived MSC; hUC-MSC: human umbilical cord-derived MSC; PEG: polyethylene glycol.

## Data Availability

The data used to support the findings of this study are available from the corresponding author upon request.
